# A Case of Consumptive Coagulopathy Before Cardiopulmonary Failure in Amniotic Fluid Embolism and Review of Literature: A Perspective of the Latent Onset and Progression of Coagulopathy

**DOI:** 10.7759/cureus.55961

**Published:** 2024-03-11

**Authors:** Tomoaki Oda, Naoaki Tamura, Daisuke Yata, Ayako Oda-Kishimoto, Toshiya Itoh, Naohiro Kubota, Yasuyuki Suzuki, Naohiro Kanayama, Hiroaki Itoh

**Affiliations:** 1 Department of Obstetrics & Gynecology, Hamamatsu University School of Medicine, Hamamatsu, JPN; 2 Department of Obstetrics and Gynecology, Fuji City General Hospital, Fuji, JPN

**Keywords:** amniotic fluid embolism (afe), disseminated intravascular coagulation (dic), induction of labor (iol), premature rupture of membranes, hemoglobin/fibrinogen ratio, pregnancy, non-reassuring fetal status, excessive fibrinolysis, critical care in obstetrics, consumptive coagulopathy

## Abstract

Amniotic fluid embolism (AFE) induces cardiopulmonary insufficiency with consumptive coagulopathy. Previous studies reported that refractory coagulopathy has already advanced at the onset of maternal cardiovascular and/or respiratory symptoms. However, when the consumption of coagulation factors starts during the clinical course, AFE remains to be elucidated. We report an intrapartum AFE case of consumptive coagulopathy before dyspnea with hypotension developing during urgent cesarean delivery that was revealed by non-reassuring fetal heart rate tracing. The patient, a 42-year-old multiparous parturient, underwent induced labor after a premature rupture of membranes in week 39 of pregnancy. Coagulation screening was initially within the normal range. Fetal heart rate monitoring demonstrated bradycardia coincided with uterine tachysystole after three hours, which required urgent cesarean section with preoperative blood screening. The hemoglobin level was maintained at 129 g/L; however, the fibrinogen value reduced to 1.79 g/L with D-dimer elevation over 60 µg/mL. Ninety minutes later, she developed dyspnea with hypotension at suturing hysterotomy. At the end of surgery, her fibrinogen further decreased to below 0.3 g/L with prolonged prothrombin time. After vigorous intensive care, she was discharged without sequelae. Consumptive coagulopathy may initiate and progress before apparent cardiopulmonary symptoms in some AFE cases. Non-reassuring fetal heart rate tracing concomitant with abrupt uterine tachysystole and/or hypertonus may be an earlier time point for the detection and intervention of AFE-related coagulopathy.

## Introduction

Amniotic fluid embolism (AFE) is a rare disease that causes severe maternal and fetal morbidity and mortality. The incidence of AFE is estimated to be five per 100,000 deliveries in Japan [1]. It is mainly characterized by abrupt onset and rapidly progressing maternal cardiopulmonary compromise that coincides with coagulopathy, leading to uncontrolled systemic hemorrhage. Previous case reports [2,3] and a study [4] reported that refractory coagulopathy with severe hypofibrinogenemia had already advanced at the onset of maternal cardiovascular and/or respiratory symptoms, such as dyspnea, hypotension, and/or cardiac arrest. We reported an AFE-induced consumptive coagulopathy with hyperfibrinolysis, which could be identified by a large discrepancy between hemoglobin (Hb) and fibrinogen (Fib) levels (Hb/Fib [H/F] ratio ≥ 100), and proposed assessments and interventions including blood transfusion based on the Japanese AFE criteria and H/F ratio [5]. However, the time point at which AFE-associated coagulopathy develops has not been investigated so far; therefore, it remains to be elucidated when coagulatory hyperactivation with the consumption of coagulation factors starts during the clinical course of AFE. It is also yet to be known if detection at an earlier stage of coagulopathy before the onset of maternal cardiopulmonary failure and/or bleeding symptoms is possible. Herein, we report an intrapartum AFE case in which consumptive coagulopathy preceded the onset of dyspnea with hypotension when fetal heart rate (FHR) monitoring showed a non-reassuring fetal heart rate (NRFHR) pattern and propose a perspective of the latent onset and progression of consumptive coagulopathy in AFE.

## Case presentation

**Figure 1:**
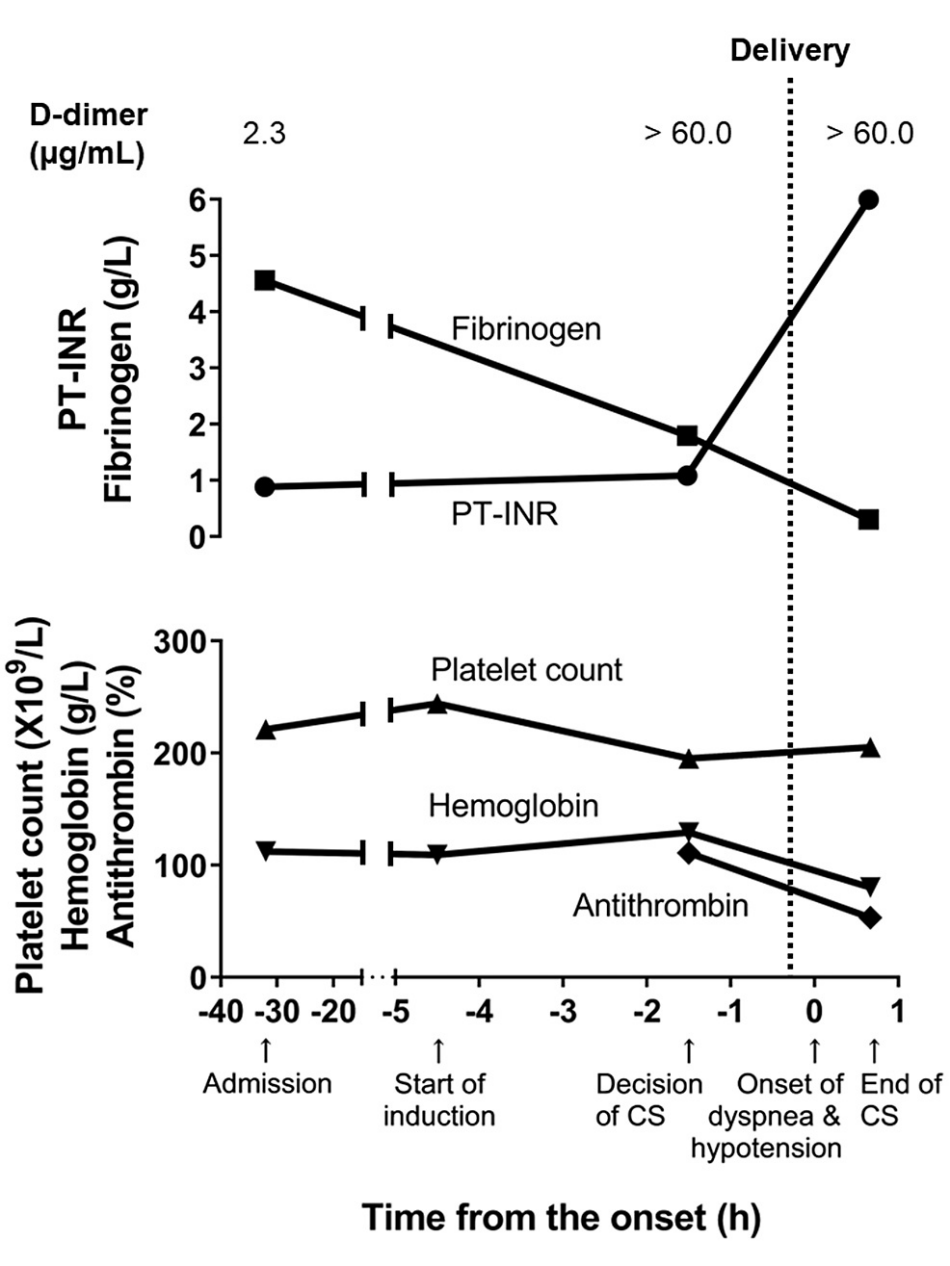
Changes in blood cell count and coagulation parameters around the onset of dyspnea with hypotension. Coagulation data were not available at the start of labor induction. The baby was born 15 minutes before the onset of dyspnea with hypotension. Abbreviations: CS, cesarean section; NRFS, non-reassuring fetal status; PT-INR, prothrombin time-international normalized ratio.

The patient, a 42-year-old woman, gravida 4, para 2, was admitted to the hospital due to premature rupture of membranes at 39 weeks of pregnancy (32 hours before the onset of dyspnea with hypotension). She had gestational hypertension (143/87 mmHg) without any anti-hypertensive drugs. She had no allergy and no family history. At admission, her blood cell counts and coagulation screening showed normal values as follows: Hb, 112 g/L; platelet count (Plt), 221 × 10^9^/L; Fib, 4.56 g/L; prothrombin time-international normalized ratio (PT-INR), 0.88; activated partial thromboplastin time (APTT), 29.0 sec; and D-dimer, 2.3 µg/mL (Figure 1). Abnormal blood cell morphology was not reported, and the serum of the blood sample drawn from the patient was not stained by hemolysis. Her chemistry laboratory screening also showed normal values as follows: aspartate aminotransferase (AST), 12 IU/L; alanine aminotransferase (ALT), 6 IU/L; lactate dehydrogenase (LDH), 165 IU/L; gamma-glutamyl transpeptidase (γ-GTP), 9 IU/L; total bilirubin, 0.4 mg/dL; urea nitrogen, 8 mg/mL; creatinine, 0.45 mg/dL; and uric acid, 4.1 mg/dL.

**Figure 2:**
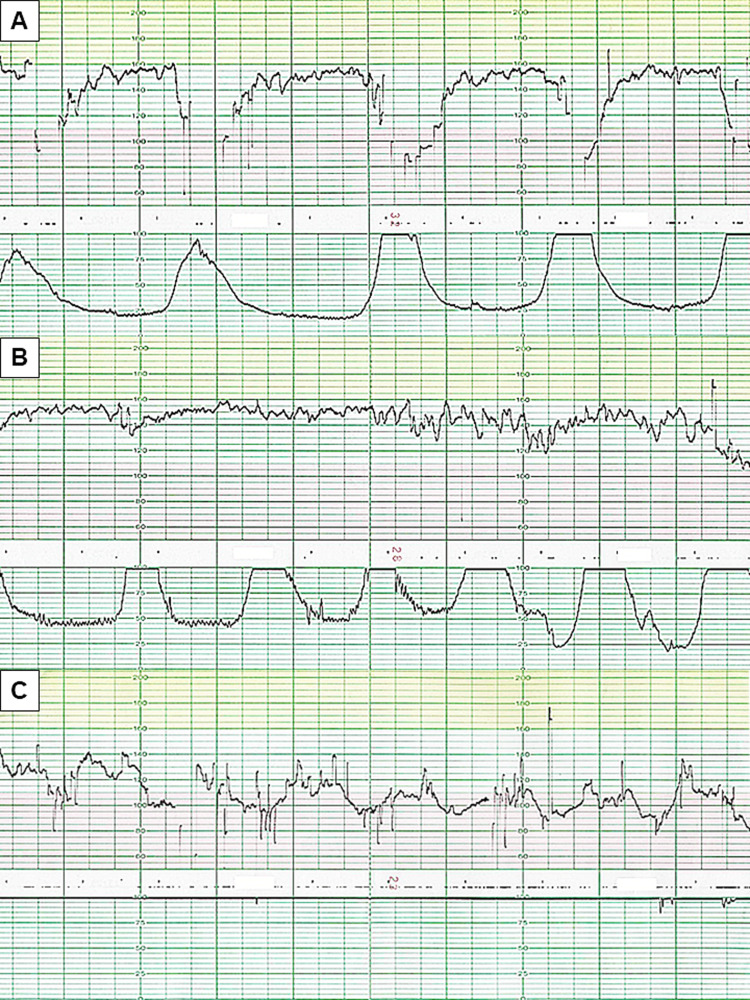
Changes in the pattern in fetal heart rate tracing during the delivery. (A) Two hours after starting labor induction (2.5 hours before the onset of dyspnea with hypotension). The FHR tracing showed mild variable deceleration. (B) 40 minutes after the tracing of (A). The chart indicated recurrent severe late deceleration with tachysystole. (C) 20 minutes after the tracing of (B). The FHR pattern showed persistent bradycardia.

Labor was induced the next morning with an oxytocin drip infusion (4.5 hours before the onset of dyspnea with hypotension). FHR demonstrated mild variable deceleration two hours later (Figure 2A), and changed to recurrent severe late deceleration with tachysystole after 40 minutes (Figure 2B). Physical examination revealed no vaginal bleeding. Ultrasonography showed the placenta located in the anterior uterine wall without any images of retroplacental hematoma. Oxytocin infusion rate decreased to half, and FHR tracing showed persistent bradycardia (90 minutes before the onset of dyspnea with hypotension) (Figure 2C).

At the same time, her blood pressure elevated to 216/55 mmHg, which was refractory to apresoline infusion. Immediately after the blood draw for preoperative screening of blood cell counts and coagulation, she underwent an operation under spinal anesthesia for termination of pregnancy and prompt fetal delivery. After the uterine lower segment incision yielded bloody amniotic fluid, she delivered a baby boy weighing 3,068 g with Apgar scores of 3 at one minute and 7 at five minutes (15 minutes before the onset of dyspnea with hypotension). The umbilical artery blood pH was 7.14. The placenta was removed with gentle traction without macroscopic evidence of placental abruption or accreta. The patient's uterine serosa showed diffuse petechial hemorrhage over the entire uterine wall. At this point, the following preoperative blood screening results were obtained: Hb, 129 g/L; Plt, 195×10^9^/L; Fib, 1.79 g/L; PT-INR, 1.08; APTT, 35.4 sec; D-dimer, > 60.0 µg/mL (Figure 1), which indicated activation of blood coagulation with the decreased Fib level. A series of serum samples of the patient was delivered to the AFE Registry Program operated by the Department of Obstetrics & Gynecology, Hamamatsu University School of Medicine, for further evaluation of the auxiliary diagnostic markers for AFE [1]. Serum zinc coproporphyrin-1 and sialyl Tn were markedly increased, which suggested amniotic fluid contamination into maternal circulation [1] (Table 1).

**Table 1:** Serum markers measured for the auxiliary diagnosis of amniotic fluid embolism. Elevated levels of zinc coproporphyrin-1 and sialyl Tn at the decision of cesarean section suggest fetal and amniotic component contamination into the maternal circulation. We could not obtain the result of the C1 esterase inhibitor at the decision of CS and all data at the end of CS due to insufficient sample amount. Abbreviations; CS, cesarean section; NA, not applicable.

Event		Admission	Decision of CS	End of CS
Time from the onset		32 h before	90 min before	45 min later
Serum marker	Reference range [1]			
Zinc coproporphyrin-1 (pmol/mL)	< 1.6	< 1.6	59.9	NA
Sialyl Tn (U/mL)	< 46	20.5	3,108	NA
Complement C3 (mg/dL)	80–140	152	204	NA
Complement C4 (mg/dL)	11–34	25.4	31.9	NA
Interleukin-8 (pg/mL)	< 20	313	242	NA
C1 esterase inhibitor (%)	≥ 42	50	NA	NA

Fifteen minutes after delivery, during suturing of the uterine incision site, the patient abruptly complained of dyspnea. Pulse oximetry (SpO_2_) showed reduced oxygen saturation to 82%. Immediate manual ventilation with 10 L/min of oxygen successfully elevated SpO_2_ to 96%, but blood pressure declined to 75/52 mmHg, and she became unresponsive within 10 minutes. Since anesthesiologists and a sufficient number of obstetricians were not available at the time of onset, the surgical assistant moved to manage the patient’s ventilation and circulation, which made it unavailable for a surgeon alone to perform a hysterectomy, stepwise devascularization, or compression suture as the treatment for atonic bleeding. The patient was intubated and diagnosed with coagulopathy with anemia at the end of the operation (45 minutes after the onset of dyspnea with hypotension): Hb, 80 g/L; Plt, 205×10^9^/L; Fib, < 0.3 g/L; PT-INR, 5.99; APTT, 132.6 sec; D-dimer, > 60.0 µg/mL (Figure 1). The platelet counts of the patient were maintained within the normal range during the operation. Arterial blood gas analysis showed acidosis with an elevated lactate level (Table 2).

**Table 2:** Physical status and arterial blood gas analysis around the onset of amniotic fluid embolism. Abbreviations: CS, cesarean section; SpO_2_, oxygen saturation by pulse oximetry; PaCO_2_, arterial partial pressure of carbon dioxide; PaO_2_, arterial partial pressure of oxygen; HCO_3_^-^, bicarbonate; NA, not available.

Event		Admission	Start of labor induction	Decision of CS	End of CS
Time from the onset		32 h before	4.5 h before	90 min before	45 min later
Physical status					
	Consciousness	Alert	Alert	Conversation possible but sick with nausea and vomit	Unresponsive to calls
	Body temperature (℃)	36.6	36.5	37.5	NA
	Blood pressure (mmHg)	135/74	125/73	216/55	65/36
	Pulse rate (/min)	98	71	93	156
	SpO_2_ (%)	NA	NA	95	98
	Oxygen administration	No	No	No	10 L/min (manual ventilation)
	Blood loss amount (mL)	0	0	0	Over 1,312
Arterial blood gas					
	pH	NA	NA	NA	7.014
	PaCO_2 _(mmHg)	NA	NA	NA	46.5
	PaO_2_ (mmHg)	NA	NA	NA	310
	Glucose (mg/dL)	NA	NA	NA	144
	HCO_3_^-^ (mEq/L)	NA	NA	NA	11.3
	Base excess (mEq/L)	NA	NA	NA	-18.0
	Lactate (mmol/L)	NA	NA	NA	11.5

**Figure 3:**
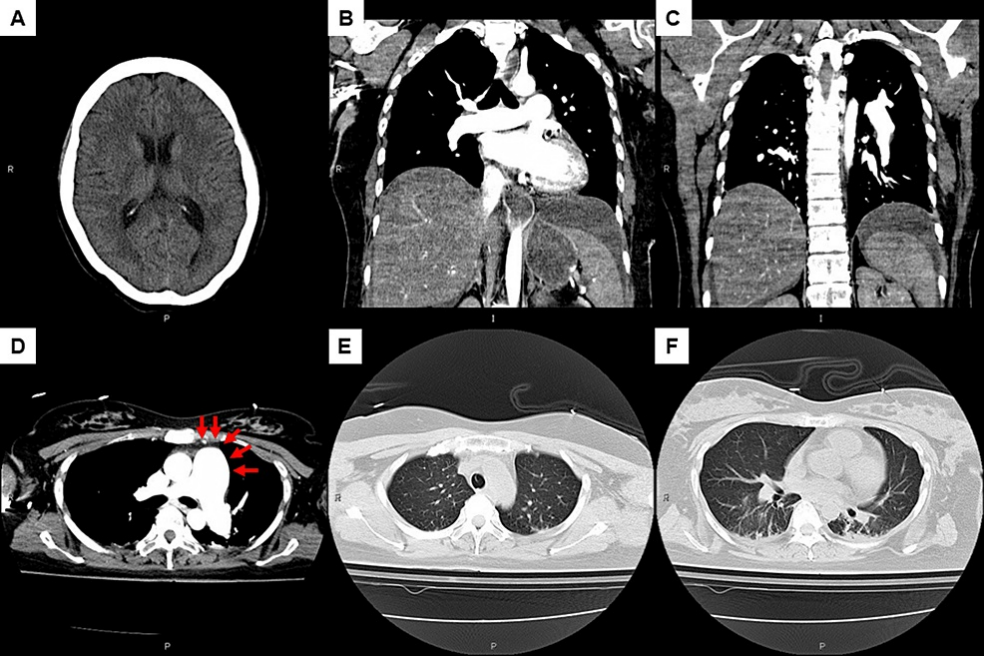
Head and chest computed tomography with contrast media one hour after the onset of dyspnea with hypotension. The patient had no evidence of intracranial hemorrhage (A), pulmonary thromboembolism (B–D), pneumothorax, pneumonia, or significant amount of pleural effusion (E, F). The pulmonary artery trunk was significantly dilated compared to the adjacent aorta (D, arrows), which suggested pulmonary hypertension.

The blood loss amount during the operation was at least 1,312 mL with unmeasured blood extensively soaked into the operating table sheets. There was no evidence of active bleeding from the uterine cavity at the end of the cesarean section; therefore, we moved to the diagnostic process and intensive management. Head and chest computed tomography did not show any evidence of intracranial hemorrhage or pulmonary thromboembolism (one hour after the onset of dyspnea with hypotension) (Figure 3).

**Figure 4:**
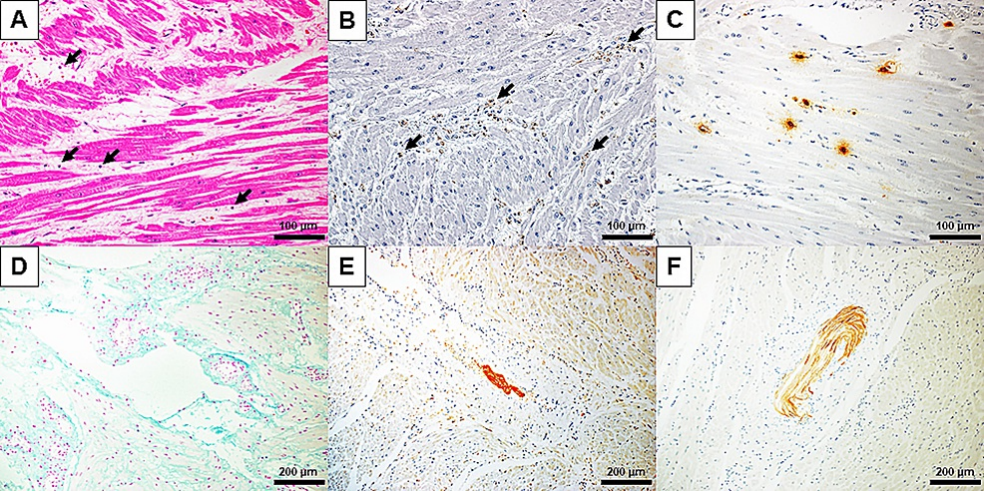
Postpartum acute myometritis with complement system activation and mast cell degranulation. Microscopic findings showed infiltration of inflammatory cells by HE staining (A, arrows) with complement C5a receptor expression (B, arrows) in uterine smooth muscles. Tryptase staining (C) showed a halo around mast cells, which indicated mast cell degranulation. Alcian blue staining (D) failed to detect fetal components in blood vessels; however, immunohistochemistry with zinc coproporphyrin-1 (E) and cytokeratin AE1/AE3 (F) showed fetus-derived constituents in uterine vessels.

In the intensive care unit, sustaining atonic bleeding refractory to uterotonics required massive red blood cells (RBCs) and fresh frozen plasma (FFP) transfusion, which was initiated immediately after the end of the cesarean section, combined with intravenous vasopressors to maintain blood pressure. During blood transfusion with manual pumping, her body temperature declined to 34.0℃. One hour after moving to the intensive care unit, the electrocardiogram monitor showed bradycardia (48 bpm) followed by cardiac arrest due to ventricular fibrillation (3.5 hours after the onset of dyspnea with hypotension), which was recovered by electrical defibrillation and a single shot of 1 mg epinephrine. Laboratory tests showed anemia (Hb, 74 g/L), thrombocytopenia (Plt, 51×10^9^/L), hypofibrinogenemia (Fib, 0.59 g/L), and metabolic acidosis despite vigorous transfusion with 40 units RBC and 68 units FFP. Persistent uterine hemorrhage, which was refractory to twice administration of 5 mg of recombinant activated factor VII, required hysterectomy and bilateral salpingo-oophrectomy to control hemorrhage (six hours after the onset of dyspnea with hypotension). The uterine tissue was soft and enlarged (weight 1,388 g) with microscopic findings of fetal components in the uterine vessels, mast cell degranulation, and infiltration of inflammatory cells expressing complement C5a receptors in the myometrium (Figure 4). The placenta was 17 × 14 × 2.2 cm in size with normal coloration on both surfaces of the fetal and maternal sides. There was no hematoma on the retroplacental area. The uninjured umbilical cord including two arteries and one vein was inserted in the center of the placenta. Histological examination did not show any evidence of infection or placental abruption.

The patient required further operations due to intraperitoneal and vaginal bleeding. Despite further RBC and FFP transfusion after hysterectomy, her anemia was not improved (Hb, 58 g/L). Contrast-enhanced computed tomography showed active bleeding into her abdominal cavity from the right inferior epigastric artery adjacent to the intraperitoneal drainage tube, which was directly sutured under laparotomy after failure in hemostasis with transcatheter arterial embolization. Over the next two days, her abdomen gradually distended due to recurrent intra-abdominal bleeding. Laparotomy revealed continuous bleeding from the omentum, the liver bed, and the vessels in the hepatoduodenal ligament, which was stopped by various techniques, including compression, cauterization, vessel-sealing devices, tissue sealing sheets, and absorbable hemostatic agents. These operations were managed by damage control surgical procedure with the temporary abdominal closure using adhesive plastic drapes, and the abdomen was finally closed on the sixth day of hysterectomy after improvement of her anemia and coagulopathy. Long-term ventilator management due to pulmonary insufficiency required a tracheotomy, and the patient was weaned from respiratory support 14 days after the hysterectomy. Vaginal hemorrhage from the stump, which was refractory to gauze compression, was successfully controlled with suturing via vaginal approach. Her total bleeding amount during the clinical course was greater than 30,000 mL and the amounts of transfused blood products were 168, 234, and 190 units for RBC, FFP, and PC, respectively, in total. She was discharged without sequelae on the 44th day from cesarean delivery.

## Discussion

The patient developed dyspnea with decreased oxygen saturation to 82% and hypotension (75/52 mmHg) 15 minutes after delivery. She had severely prolonged PT-INR (5.99) and a decreased fibrinogen level (<0.3 g/L) with normal platelet counts at the end of the cesarean section. She did not have a fever or retroplacental hematoma during her clinical course. Therefore, we excluded placental abruption from the differential diagnosis, and this case was diagnosed as AFE by both the Japanese AFE diagnostic criteria (Table 3) [1] and those proposed by Clark (Table 4) [6]. The microscopic findings of postpartum acute myometritis (Figure 4) may cause atonic bleeding refractory to uterotonics [7-9]. 

**Table 3:** Japanese diagnostic criteria for amniotic fluid embolism (AFE). Because these diagnostic criteria serve the purpose of making a clinical diagnosis and being able to promptly provide treatment, the pathological conditions that meet them may include those other than AFE. Abbreviation: AFE: amniotic fluid embolism. Citation: [1]

Diagnostic criteria	
(1)	If symptoms appeared during pregnancy or within 12 h of delivery.
(2)	If any intensive medical intervention was conducted to treat one or more of the following symptoms/diseases:
	A) Cardiac arrest
	B) Severe bleeding of unknown origin within 2 hours of delivery (≥ 1500 mL)
	C) Disseminated intravascular coagulation
	D) Respiratory failure
(3)	If the findings or symptoms obtained cannot be explained by other diseases.
A clinical diagnosis of AFE can be made if the pathological condition meets the above three criteria.	

**Table 4:** Clark’s diagnostic criteria for amniotic fluid embolism (AFE). The scoring system for the diagnosis of overt DIC involves the platelet count (> 100,000/µL = 0, < 100,000/µL = 1, < 50,000/µL = 2), prolonged prothrombin time or international normalized ratio (< 25% increase = 0, 25–50% increase = 1, > 50% increase = 2), and fibrinogen level (> 2.0 g/L = 0, < 2.0 g/L = 1), with a combined score of 3 or more indicating overt DIC. Abbreviations: AFE: amniotic fluid embolism, DIC: disseminated intravascular coagulation. Citation: [6]

Diagnostic criteria	
1.	Sudden onset of cardiorespiratory arrest, or both hypotension (systolic blood pressure < 90 mm Hg) and respiratory compromise (dyspnea, cyanosis, or peripheral capillary oxygen saturation [SpO_2_] < 90%).
2.	Documentation of overt DIC following the appearance of these initial signs or symptoms, using the scoring system of the Scientific and Standardization Committee on DIC of the ISTH, modified for pregnancy. Coagulopathy must be detected prior to loss of sufficient blood to account for dilutional or shock-related consumptive coagulopathy.
3.	Clinical onset during labor or within 30 min of delivery of placenta.
4.	No fever (≥ 38.0°C) during labor.

In this AFE case, coagulatory hyperactivation with the decreased fibrinogen level, which might cause bloody amniotic fluid and diffuse petechial hemorrhage on the uterine serosa, already existed before her clinical manifestation of dyspnea with hypotension. If the laboratory screening for CBC and coagulation had not been tested just before the cesarean section, we would have only obtained those data presenting advanced coagulopathy evaluated at the end of the cesarean section and lost the opportunity to know consumptive coagulopathy had already started before the onset of dyspnea with hypotension. We performed a literature search for English articles that described cases and original investigations involving AFE that were diagnosed with the criteria proposed by Clark [6] and reviews/guidelines of AFE that included a clear statement of a chronological relationship between cardiopulmonary symptoms and evaluation of coagulopathy in PubMed up to March 2023 using the search terms “amniotic fluid embolism” and “coagulopathy.” We retrieved 19 case reports/series [10-28], five original articles [5,29-32], and 11 reviews/guidelines [33-43] (Table 5). The original articles and case reports/studies reported that AFE-associated coagulopathy was evaluated and detected after cardiopulmonary symptoms, which was supported by the reviews reporting that it developed after cardiorespiratory insufficiency. Thus, this is the first case described clearly as consumptive coagulopathy that was detected before cardiopulmonary symptoms to the best of our knowledge.

**Table 5:** Previous reports that described the timepoint of coagulopathy in amniotic fluid embolism diagnosed with Clark’s criteria. ^1^ Case number only includes cases that meet the diagnostic criteria proposed by Clark et al. [6]. ^2^ The variables are described as the medians with ranges from minimum to maximum along with the description in the references. Abbreviations: NRFHR, non-reassuring fetal heart rate; NA, not applicable; AFE, amniotic fluid embolism. Citation: [6]

(A) Case reports/series						
Author (Year)	Case number^1^	Age (year)	Gestational age (week)	Initial clinical presentation	Isolated NRFHR tracing before clinical presentation	Timepoint of coagulopathy initially evaluated
Oda et al. (This case)	1	42	39	Dyspnea with hypotension followed by cardiac arrest	Yes	Before clinical presentation
Oliver et al. (2022) [10]	1	36	39	Impaired consciousness followed by cardiac arrest	No	After clinical presentation
Wothe et al. (2022) [11]	1	29	39	Impaired consciousness followed by cardiac arrest	Yes	After clinical presentation
Li et al. (2022) [12]	1	27	39	Abdominal pain, convulsion, and dyspnea with hypotension	No	After clinical presentation
Simard et al. (2021) [13]	1	38	40	Hypotension followed by cardiac arrest	NA	After clinical presentation
Gitman et al. (2019) [14]	1	42	34	Cardiac arrest	No	After clinical presentation
Loughran et al. (2019) [15]	1	21	NA	Disoriented followed by cardiac arrest	Yes	After clinical presentation
Hurwich et al. (2016) [16]	1	35	Term	Respiratory distress followed by cardiac arrest	NA	After clinical presentation
Buechel et al. (2015) [17]	1	29	38	Syncope followed by hypoxia with hypotension	NA	After clinical presentation
Rogers et al. (2013) [18]	1	27	36	Cardiac arrest	No	After clinical presentation
Belfort et al. (2011) [19]	1	33	36	Hypoxia followed by cardiac arrest	NA	After clinical presentation
Liao et al. (2011) [20]	2	36	NA	Dyspnea with hypotension	NA	After clinical presentation
		35	37	Cardiac arrest	NA	After clinical presentation
Mato et al. (2008) [21]	1	40	41	Impaired consciousness and dyspnea with hypotension	No	After clinical presentation
Peitsidou et al. (2008) [22]	1	29	40	Cyanosis, hypotension, seizure, and cardiac arrest	Yes	After clinical presentation
Stehr et al. (2007) [23]	1	28	39	Unconsciousness and cyanosis followed by cardiac arrest	No	After clinical presentation
McDonnell et al. (2007) [24]	1	35	41	Dyspnea and seizure followed by cardiac arrest	NA	After clinical presentation
Robillard et al. (2005) [25]	1	30	31	Hypotension followed by cardiac arrest	NA	After clinical presentation
Lim et al. (2004) [26]	1	26	39	Respiratory failure with hypotension	Yes	After clinical presentation
Fletcher et al. (2000) [27]	1	41	41	Hypoxia with hypotension	Yes	After clinical presentation
Sprung et al. (1992) [28]	1	27	42	Dyspnea and seizure followed by cardiac arrest	NA	After clinical presentation
(B) Original research						
Author (Year)	Case number^1^	Age (year)	Gestational age (week)	Clinical presentation	Isolated NRFHR tracing before clinical presentation	Timepoint of coagulopathy evaluated
Aissi et al. (2022) [29]	10	34 (24–40)^2^	36 (30–41)^2^	Cardiac arrest (n = 7), Cardiovascular collapse (n = 9), Acute respiratory failure (n = 3)	NA	After clinical presentation
Kim et al. (2021) [30]	4	32 (31–37)^2^	39 (23–40)^2^	Hypotension (n = 1), Tingling sensation of limbs (n = 1), Agitation/Dyspnea (n = 1), Vaginal bleeding (n = 1)	NA	After clinical presentation
Oda et al. (2020) [5]	6	34 (27–42)^2^	39 (29–41)^2^	Respiratory compromise with hypotension (n = 2), Cardiopulmonary arrest (n = 4), Postpartum hemorrhage (n = 1), Uterine atony (n = 1)	NA	After clinical presentation
Schröder et al. (2020) [31]	3	32 (28–36)^2^	39 (38–41)^2^	Ventricular extrasystoly, impaired consciousness, and bleeding (n = 1), Impaired consciousness with vomiting and bleeding (n = 1), Cardiorespiratory symptoms and vaginal bleeding (n = 1)	No	After clinical presentation
Bonnet et al. (2018) [32]	21	> 35 (n = 9), ≤ 35 (n = 12)	< 37 (n = 3), ≥ 37 (n = 18)	Cardiopulmonary collapse (n = 21), Clinical coagulopathy (n = 20), Respiratory deficiency (n = 11), Seizure (n = 5)	Yes (n = 4)	After clinical presentation
(C) Reviews and guidelines						
Author (Year)	Description on the timepoint of the coagulopathy in AFE					
Haftel et al. (2023) [33]	“Disseminated intravascular coagulation (DIC) occurs in approximately 80% of patients with AFE. This may be immediate at the time of the cardiopulmonary collapse or delayed. This may be immediate at the time of the cardiopulmonary collapse or delayed.”					
Coggins et al. (2022) [34]	“AFE should be suspected in any laboring or recently postpartum patient who presents with sudden-onset hypoxia and hypotension, particularly if rapidly followed by coagulopathy.”					
Royal College of Obstetricians & Gynaecologists (2019) [35]	“Coagulopathy often develops if the mother survives long enough, often giving rise to massive postpartum haemorrhage.”					
Sundin et al. (2017) [36]	“The diagnosis is based on clinical status when the classic triad of hypoxia, hypotension, and subsequent coagulopathy are noted in a laboring woman or woman who just gave birth, and no other plausible explanation can be determined.”					
Shamshirsaz et al. (2016) [37]	“In its classic form, a woman in labor or shortly after vaginal or cesarean delivery sustains acute dyspnea, desaturation, or dyspnea followed by sudden cardiovascular collapse or arrest. These symptoms are commonly followed by the development of coagulopathy (83% of patients).”					
Kaur et al. (2016) [38]	“The onset can occur as quickly as 10-30 min from the onset of symptoms or may be delayed by as long as 4 h.”					
Sultan et al. (2016) [39]	“Laboratory tests should be repeated frequently in the initial phase of AFE presentation as coagulopathy may develop in the first few hours.”					
Society for Maternal-Fetal Medicine (2016) [40]	“The typical presentation of amniotic fluid embolism includes a triad of sudden hypoxia and hypotension, followed in many cases by coagulopathy”					
Thongrong et al. (2013) [41]	“Coagulopathy may be one of the most prominent signs of AFE, it usually appears within 4 hours of the initial presentation.”					
Gilmore et al. (2003) [42]	“Of the patients who survive the initial hemodynamic collapse, 70% develop noncardiogenic pulmonary edema that resembles acute respiratory distress syndrome on chest radiograph. Forty-five percent develop a severe coagulopathy, disseminated intravascular coagulation (DlC), within 30 minutes to 4 hours.”					
Lau et al. (1994) [43]	“Recent evidence points towards a combination of a severe haemodynamic disturbance, consisting of transient pulmonary hypertension, profound hypoxia and left ventricular failure, followed by secondary coagulopathy in about 40 per cent of patients who survive the initial event.”					

**Figure 5:**
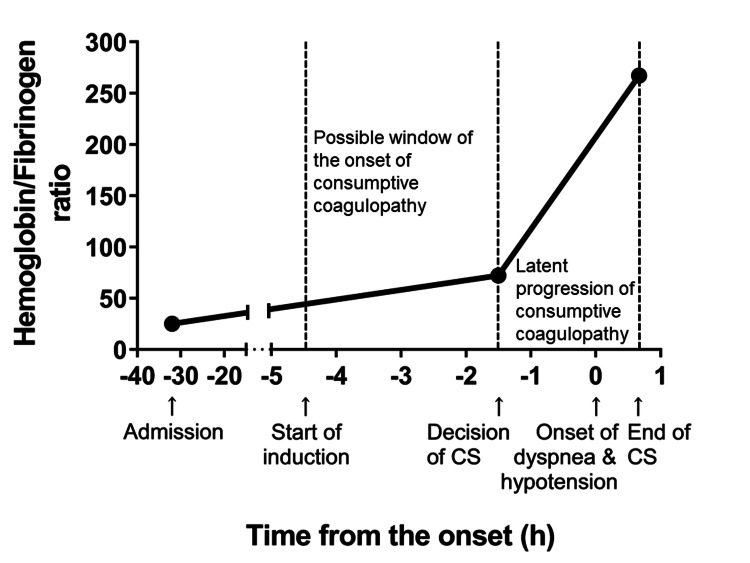
Changes in the hemoglobin/fibrinogen (H/F) ratio around the onset of dyspnea with hypotension. The activation of blood coagulation with a decrease in fibrinogen level might start at a particular time point between labor induction and the decision of CS, which increased the H/F ratio to 72 at the decision of CS; furthermore, it increased latently to 267 at the end of surgery. Abbreviation: CS, cesarean section.

We propose a preemptive strategy that encourages AFE to be clinically differentiated based on the Japanese AFE diagnostic criteria, which allows for moving to treatment immediately when patients have cardiopulmonary failure and/or postpartum hemorrhage during labor or a few hours after delivery. Furthermore, coagulation factors should be replaced promptly if the patient’s H/F ratio is more than 100 [5]. In this case, the H/F ratio increased to more than 100 at the end of surgery after the onset of dyspnea and hypotension; however, it had already elevated to 72 with decreased Fib and increased D-dimer levels at the decision of urgent cesarean section, 90 minutes before the onset of the symptoms (Figure 5).

This change in coagulation parameters and H/F ratio during delivery supports the hypothesis that Fib decrease due to consumptive coagulopathy may start before cardiopulmonary failure with an initial H/F ratio <100, followed by progressive hypofibrinogenemia increasing the H/F ratio over 100, which would be usually recognized after developing cardiovascular and/or respiratory symptoms. If coagulation screening is performed before the development of cardiopulmonary symptoms, the initiation and/or aggravation of consumptive coagulopathy may be revealed and treated earlier.

In this case, the laboratory data of the preoperative CBC and coagulation screening at the time we decided on the urgent cesarean section due to NRFHR tracing showed coagulatory hyperactivation with a decrease in the fibrinogen level, i.e., consumptive coagulopathy. It has been reported that electronic FHR monitoring often demonstrates NRFHR patterns with decelerations, loss of variability, and bradycardia in intrapartum AFE cases, possibly due to insufficient oxygenated blood supply, which could be aggravated by hypertonic uterine state [40]. Moreover, such FHR abnormalities commonly accompany or even precede apparent maternal signs and symptoms [44,45]. Our previous studies showed fibrin clots in uterine vessels and platelet-rich microthrombi in pulmonary vessels in AFE autopsy cases [46] and excessive fibrinolytic activation with the increase and decrease in plasma tissue plasminogen activator and thrombin activatable fibrinolysis inhibitor, respectively [47]. Based on these findings, we speculate that fibrin clots produced in uterine vasculature due to coagulation activation might cause consumption of coagulation factors and hypoperfusion and hypoxia in the uterine tissue, which could be aggravated with ventilation-perfusion mismatch in pulmonary circulation, resulting in fetal hypoxia presenting NRFHR tracing. The hypoxic change might also activate the maternal fibrinolytic system, leading to consumptive coagulopathy with hyperfibrinolysis. Thus, we suppose tissue hypoxia and hypoperfusion might be a common underlying mechanism that indirectly associates NRFHR tracing with coagulopathy involving AFE.

Although it has not been fully clarified which pathological changes cause NRFHR patterns and coagulopathy and whether there is a direct association between them, we suggest NRFHR tracing might be one of the few time points that trigger earlier detection of consumptive coagulopathy preceding respiratory and/or cardiovascular symptoms. Since the NRFHR pattern is so common in obstetric practice while the incidence of AFE is rare, it is not practical to consider the possible development of AFE in all cases. Nonetheless, based on the previous reports that described uterine tachysystole in conjunction with NRFHR before maternal cardiovascular collapse [40,45] and the present case, we speculate that NRFHR tracing complicated with abrupt uterine tachysystole and/or hypertonus during delivery may be a sign of AFE prior to recognizable maternal cardiopulmonary symptoms in at least a part of the maternal cases presenting such tracing. Other obstetric diseases that can develop consumptive coagulopathy include placental abruption, which should be ruled out with ultrasound findings and placental pathology [48]. If the parturient has decreased Fib and increased D-dimer levels with an elevation of H/F ratio (even if below 100) when NRFHR tracing with uterine tachysystole and/or hypertonus is observed during delivery, physicians should consider the possible development of AFE; prepare blood transfusion with enough amount of FFP, cryoprecipitate, fibrinogen concentrate, and anti-fibrinolytic agents as suggested in the previous reports [47,49,50]; and monitor the onset of cardiopulmonary symptoms and progression of coagulopathy closely. This preemptive intervention may lead to a better maternal prognosis.

## Conclusions

Consumptive coagulopathy in the AFE case preceded the onset of dyspnea with hypotension by 90 minutes and aggravated hypofibrinogenemia latently until the appearance of those symptoms. Although we still do not know when the origin of coagulatory activation with consumption of coagulation factors arises in AFE and what causes it, we have shown here that AFE-associated coagulopathy may already be in place and progressing before the appearance of cardiorespiratory symptoms in at least a part of AFE cases. NRFHR tracing with abrupt uterine tachysystole despite no apparent retroplacental hematoma may be one of the earlier time points to detect preceding consumptive coagulopathy involving intrapartum AFE, which may enable preemptive intervention.
